# Spousal Support and Physician Work-Life Integration and Burnout

**DOI:** 10.1001/jamanetworkopen.2025.9507

**Published:** 2025-05-09

**Authors:** Ciara C. O’Sullivan, Sarah Jenkins, Andrea N. Leep Hunderfund, Kathryn J. Ruddy, Colin P. West, Ariela L. Marshall

**Affiliations:** 1 Department of Oncology, Mayo Clinic, Rochester, Minnesota; 2 Department of Quantitative Health Sciences, Mayo Clinic, Rochester, Minnesota; 3 Department of Neurology, Mayo Clinic, Rochester, Minnesota; 4 Division of General Internal Medicine, Department of Medicine, Mayo Clinic, Rochester, Minnesota; 5 Division of Hematology, Oncology, and Transplantation, University of Minnesota, Minneapolis

## Abstract

**Importance:**

Few studies have assessed whether factors outside the workplace, such as support from a spouse or partner, are associated with work-life integration (WLI) satisfaction and burnout among physicians. Evaluating such an association may help with developing novel strategies to address burnout and improve WLI satisfaction among this group.

**Objectives:**

To compare spousal support among female and male physicians and explore associations of spousal support with WLI satisfaction and burnout.

**Design, Setting, and Participants:**

This cross-sectional, multispecialty survey study was conducted among 661 faculty physicians employed at an academic medical center from March 26 to May 22, 2024.

**Main Outcomes and Measures:**

Survey items measured spousal support (5 items), WLI satisfaction (1 item), and burnout (2 Maslach Burnout Inventory items). Associations of spousal support and gender with WLI and burnout were assessed using logistic regression analyses adjusting for age, race and ethnicity, weekly work hours, and weekly hours spent on household or childcare duties.

**Results:**

Overall, 739 of 2103 faculty physicians (response rate, 35.1%) completed the survey, of whom 661 (89.4%) were currently married or partnered (359 of 661 men [54.3%]). In this subset, 322 of 351 male physicians (91.7%) and 276 of 298 of female physicians (92.6%) reported frequent career support (defined as often, most of the time, or always) over the last year from their spouse or partner. Physicians reporting high levels of career support from their spouse or partner had higher odds of WLI satisfaction (odds ratio [OR], 1.50 [95% CI, 1.22-1.86]; *P* < .001) and lower odds of burnout (OR, 0.73 [95% CI, 0.61-0.87]; *P* < .001), independent of gender and adjusted for age, race and ethnicity, weekly work hours, and weekly hours spent on household or childcare duties. Male physicians had higher odds of WLI satisfaction than female physicians (OR, 1.95 [95% CI, 1.32-2.90]; *P* < .001), even after adjusting for spousal career support and other covariates listed, whereas odds of burnout did not significantly differ by gender (OR, 0.84 [95% CI, 0.57-1.23]; *P* = .37).

**Conclusions and Relevance:**

This study suggests that the level of career support from a spouse or partner is associated with WLI satisfaction and burnout among physicians, independent of gender. Future studies should explore whether interventions designed to enhance career support can improve WLI and well-being among physicians.

## Key Points

**Question:**

Is support from a spouse or partner associated with work-life integration (WLI) and burnout among physicians?

**Findings:**

In this cross-sectional survey study that included 661 married or partnered faculty physicians, respondents reporting high levels of career support from their spouse or partner had higher odds of WLI satisfaction and lower odds of burnout. Male physicians had higher odds of WLI satisfaction than female physicians, whereas odds of burnout did not statistically differ by gender.

**Meaning:**

These findings suggest that the level of career support from a spouse or partner is associated with WLI satisfaction and burnout among physicians, regardless of gender.

## Teaser

This cross-sectional survey study compares spousal support among female and male physicians and explores associations of spousal support with work-life integration satisfaction and burnout.

## Introduction

Distress among physicians has reached dangerous levels, with recent data suggesting that more than 60% of US physicians experience symptoms of burnout.^1^ Burnout is a work-related experience characterized by emotional exhaustion, cynicism (depersonalization), and perceived low levels of personal accomplishment.^2^ Adverse consequences may include reduced professionalism, decreased patient care quality, increased medical errors, and early retirement.^3^ Furthermore, only 30% of physicians report satisfaction with work-life integration (WLI).^1^ Work-life integration is achieved when a physician’s career and personal responsibilities are balanced and is associated with lower stress, increased productivity, and improved employee retention^4,5^

Individual and institutional factors associated with WLI satisfaction and burnout are well described.^6^ However, few studies have explored how factors extrinsic to the workplace, such as support from a spouse or partner (referred to hereafter as spousal support) and caregiving responsibilities outside work, are associated with physician WLI and burnout. Although support from colleagues and friends can mitigate work-related stress,^7,8,9^ spousal support remains one of the most effective sources for navigating work-related stress.^9,10,11^ Potential benefits also include support of career goals, assistance with childcare and household duties, and emotional and financial support.^12,13^ Although some studies have evaluated links between spousal support and WLI and burnout, existing research is dated, limited by small sample size, or focused only on dual-physician marriages.^13,14,15,16^ To address these limitations, we undertook a cross-sectional survey of faculty physicians at a large, US academic health center to (1) characterize perceived frequency of spousal support among physicians and (2) explore associations of spousal support and gender with WLI satisfaction and burnout, adjusting for age, race and ethnicity, weekly work hours, and weekly hours spent on household tasks or childcare.

## Methods

### Study Design and Participants

We distributed an institutional survey link via email to a list of 2103 faculty consultants employed at Mayo Clinic in Rochester, Minnesota, using Qualtrics software (2024 Qualtrics) from March 26 to May 22, 2024 (eAppendix in Supplement 1). In addition to the email invitation, there was also an announcement with a QR code for the survey displayed in a faculty lounge and in an institutional physician faculty newsletter. The sample size (n = 2103) for survey distribution was determined from a list of potentially eligible faculty physicians provided by the Mayo Clinic human resources department; no additional sampling was performed.

This study was conducted in accordance with the Declaration of Helsinki^17^ and the study protocol was reviewed and exempted by the Mayo Clinic institutional review board. Written informed consent was obtained from all participants prior to study completion. This study followed the Strengthening the Reporting of Observational Studies in Epidemiology (STROBE) reporting guideline.

### Data Collection

Recruitment emails and the screen-based announcement described the study purpose (ie, to better understand how spousal support is associated with WLI and burnout) and emphasized that participation was voluntary. Emails included a link to an online consent form (accessible via QR code); physicians who consented could then access the survey. We sent up to 3 email reminders to eligible physicians who had not yet responded.

### Study Measures

We measured spousal support using 5 items derived from the Support in Intimate Relationships Rating Scale^18^: “Over the past one year, my spouse/partner has been supportive of my career,” “Whenever I am busy with professional and household responsibilities, my spouse/partner offers to help me (eg, offers to do my chores),” “Whenever I am busy with professional and household responsibilities, my spouse/partner helps me (eg, does my chores),” “When I have a problem at work, my spouse/partner expresses confidence in my ability to handle a situation,” and “When I have a problem at work, my spouse/partner takes my side when discussing my situation.” Response options measured perceived frequency of support over the past year on a scale of 0 (never) to 5 (always). We calculated a composite spousal support score (range, 0-5) by averaging responses to these 5 items.

We measured WLI using a single item, “My work schedule leaves me enough time for my personal/family life,” with response options of strongly agree, agree, neutral, disagree, or strongly disagree. Individuals who selected strongly agree or agree were deemed satisfied with WLI, in keeping with prior large surveys of physicians.^19^

We measured burnout using a 2-item screening assessing emotional exhaustion and depersonalization, with burnout defined as experiencing at least weekly symptoms for either or both domains. These burnout items were derived from the Maslach Burnout Inventory, a validated 22-item instrument considered the criterion standard for measuring burnout, used under license from Mind Garden Inc. This approach has previously been used in other large studies evaluating physician burnout.^20,21,22^ Additional survey items measured relationship status (single, married, partnered, separated or divorced, widowed or widower); demographic information (age, gender, race and ethnicity, specialty, years in practice, and full-time equivalent status); number and ages of children (if applicable); if the respondent acted as a caregiver for other children or an individual who was older, unwell, and/or had special needs; hours per week spent on work duties; hours per week spent on household or childcare duties; and access to friends, family, and/or external professional services to assist. Race and ethnicity were self-reported, and we classified physicians who identified as American Indian or Alaska Native, Black or African American, Hispanic or Latina/o/x, and Native Hawaiian or Other Pacific Islander races and ethnicities as being underrepresented in medicine (URM) based on the Association of American Medical Colleges Medical Minority Applicant Registry definition.^23^ Those reporting nonspecified other races were not included due to insufficient information to classify them as URM or not.

### Statistical Analysis

We calculated the survey response rate among the list of eligible physicians using the American Association for Public Opinion Research response rate 1 definition, defining a complete response as one in which 50% or more of applicable items were answered (partial responders with <50% of items completed were included only in the denominator).^24^ No imputation was used for missing data among survey completers, and all denominators reflect those with available data (which may vary across variables). Gender, age group, and race and ethnicity (URM vs non-URM) were compared between survey completers and aggregated data from the full set of eligible physicians (N = 2103) with 1-way χ^2^ goodness-of-fit tests. Analyses were restricted to respondents who were currently married or partnered, identifying as male or female. Basic summary statistics were reported (limiting subgroup analyses to groups with ≥5 respondents to protect anonymity). Unadjusted comparisons by group (ie, males vs females) were assessed with χ^2^ tests for categorical data or with Kruskal-Wallis tests for continuous or ordinal data (eg, for the 6-point scale spousal support items).

Next, we used logistic regression to examine associations of gender and spousal support with burnout and WLI, adjusting for age, race and ethnicity (categorized as URM or non-URM), weekly work hours, and weekly hours spent on household or childcare duties. We included these covariates because they were associated with either an outcome (WLI or burnout) or gender in univariate analyses or prior literature, while avoiding the inclusion of highly correlated covariates (eg, age and years in practice, weekly work hours and full-time equivalent status) or survey items applicable only to a subset of respondents (eg, age of children applicable to only parents, spouse’s work hours applicable only to those working). Adjusting for gender and covariates associated with gender was important to help adjust for any potential response bias by gender. We conducted a series of 6 multivariable logistic models for each outcome, 1 model for each spousal support item (models 1-5) and another for the composite spousal support score (model 6). The spousal support items and composite score were each included in the models as continuous variables (ie, they were not dichotomized). Interactions between spousal support and gender were investigated. Odds ratios (ORs) and 95% CIs were reported. All *P* values were from 2-sided tests and results were deemed statistically significant at *P* < .05. All analyses were performed using R, version 4.2.2 (R Project for Statistical Computing).

## Results

Overall, 739 of 2103 eligible physicians completed the survey (response rate, 35.1%). Reasons for survey nonresponse were unknown. Analyses were restricted to 661 respondents (89.4%) identifying as male or female who were married or partnered. A total of 359 (54.3%) of the physicians were male and 302 (45.7%) were female. Individuals with other or nonspecified gender identity (16 of married or partnered respondents) were not included due to insufficient sample size. Table 1^23^ summarizes demographics of this subset of respondents. Of 661 married or partnered respondents, 38 (5.7%) were of Hispanic, Latina/o/x, or Spanish origin, 58 (8.8%) were Asian, 12 (1.8%) were Black or African American, 507 (76.7%) were White, 17 (2.6%) were of other race, 8 (1.2%) were of multiple races, and 21 (3.2%) did not provide their race or ethnicity. Most respondents were 40 to 49 years of age (217 of 660 [32.9%]) or 50 to 59 years of age (171 of 660 [25.9%]), 53 of 618 (8.6%) identified their race or ethnicity as URM, and 261 of 661 (39.5%) had been in practice for 20 years or more. Male respondents were older than the female respondents (112 of 358 men [31.3%] vs 27 of 302 [8.9%] women were aged ≥60 years; *P* < .001). Multiple medical specialties were represented, with the most commonly reported being internal medicine and other medical subspecialties (245 of 660 [37.1%]). Compared with the full group of eligible physicians (N = 2103), distribution of age and race and ethnicity was similar among survey completers. Females were more heavily represented among survey completers compared with the full group (302 of 661 [45.7%] vs 749 of 2103 [35.6%]; *P* < .001).

**Table 1.** Characteristics of Married or Partnered Survey Respondents

Characteristic	No./total No. (%)			*P* value
	Male (n = 359 [54.3%])	Female (n = 302 [45.7%])	Total (N = 661)	
Age, y				
30-39	48/358 (13.4)	85/302 (28.1)	133/660 (20.2)	<.001
40-49	109/358 (30.4)	108/302 (35.8)	217/660 (32.9)	
50-59	89/358 (24.9)	82/302 (27.2)	171/660 (25.9)	
≥60	112/358 (31.3)	27/302 (8.9)	139/660 (21.1)	
Underrepresented race and ethnicity in medicine^a^				
No	315/331 (95.2)	250/287 (87.1)	565/618 (91.4)	<.001
Yes	16/331 (4.8)	37/287 (12.9)	53/618 (8.6)	
Years in practice				
<10	78/359 (21.7)	107/302 (35.4)	185/661 (28.0)	<.001
10-19	99/359 (27.6)	116/302 (38.4)	215/661 (32.5)	
≥20	182/359 (50.7)	79/302 (26.2)	261/661 (39.5)	
Specialty				
Allergy and immunology	4/358 (1.1)	1/302 (0.3)	5/660 (0.8)	NA^b^
Anesthesiology	29/358 (8.1)	18/302 (6.0)	47/660 (7.1)	
Colon and rectal surgery	1/358 (0.3)	3/302 (1.0)	4/660 (0.6)	
Dermatology	4/358 (1.1)	11/302 (3.6)	15/660 (2.3)	
Emergency medicine	10/358 (2.8)	9/302 (3.0)	19/660 (2.9)	
Family medicine	11/358 (3.1)	15/302 (5.0)	26/660 (3.9)	
Internal medicine and other medicine subspecialties	136/358 (38.0)	109/302 (36.1)	245/660 (37.1)	
Medical genetics and genomics	0	0	0	
Neurologic surgery	3/358 (0.8)	1/302 (0.3)	4/660 (0.6)	
Neurology	18/358 (5.0)	9/302 (3.0)	27/660 (4.1)	
Nuclear medicine	1/358 (0.3)	1/302 (0.3)	2/660 (0.3)	
Obstetrics and gynecology	4/358 (1.1)	9/302 (3.0)	13/660 (2.0)	
Ophthalmology	4/358 (1.1)	3/302 (1.0)	7/660 (1.1)	
Orthopedic surgery	8/358 (2.2)	2/302 (0.7)	10/660 (1.5)	
Pathology	14/358 (3.9)	19/302 (6.3)	33/660 (5.0)	
Pediatrics	22/358 (6.1)	34/302 (11.3)	56/660 (8.5)	
Physical medicine and rehabilitation	12/358 (3.4)	5/302 (1.7)	17/660 (2.6)	
Plastic surgery	1/358 (0.3)	1/302 (0.3)	2/660 (0.3)	
Preventive medicine	1/358 (0.3)	1/302 (0.3)	2/660 (0.3)	
Psychiatry	12/358 (3.4)	7/302 (2.3)	19/660 (2.9)	
Radiation oncology	9/358 (2.5)	4/302 (1.3)	13/660 (2.0)	
Radiology	29/358 (8.1)	18/302 (6.0)	47/660 (7.1)	
Surgery and other surgical subspecialties	20/358 (5.6)	18/302 (6.0)	38/660 (5.8)	
Thoracic surgery	0	0	0	
Urology	5/358 (1.4)	4/302 (1.3)	9/660 (1.4)	

Abbreviation: NA, not applicable.

^a^ We classified physicians who identified as American Indian or Alaska Native, Black or African American, Hispanic or Latina/o/x, and Native Hawaiian or Other Pacific Islander races and ethnicities as being underrepresented in medicine based on the Association of American Medical Colleges Medical Minority Applicant Registry definition.^23^

^b^ Specialty distribution was not statistically compared between groups because of the high number of categories.

Table 2 summarizes work and life experiences of male and female physician respondents. Males reported more weekly work hours than females (270 of 356 [75.8%] vs 193 of 299 [64.5%] working ≥50 hours per week; *P* = .01) and were more likely to work full time (281 of 352 [79.8%] vs 184 of 300 [61.3%]; *P* < .001). Most respondents’ spouses (444 of 650 [68.3%]) had a paid job, with 179 of 650 (27.5%) also working as physicians. Of employed spouses, 144 of 442 (32.6%) worked part time and 132 of 444 (29.7%) worked a mean of 50 hours or more per week. Spousal employment differed statistically significantly by gender, with male respondents being less likely than female respondents to have a physician spouse (69 of 352 [19.6%] vs 110 of 298 [36.9%]) and more likely to have a spouse without a paid job (152 of 352 [43.2%] vs 54 of 298 [18.1%]; *P* < .001). Male respondents were also less likely than female respondents to report that their spouse worked full time professionally (95 of 200 [47.5%] vs 203 of 242 [83.9%]; *P* < .001). Nearly 9 in 10 respondents (586 of 661 [88.7%]) had children (median, 2 [range, 1-8]), and 130 of 586 (22.2%) of parents noted their youngest child was younger than 5 years.

**Table 2.** Work and Life Experiences of Married or Partnered Survey Respondents

Variable	No./total No. (%)			*P* value
	Male (n = 359)	Female (n = 302)	Total (N = 661)	
Work experiences				
Mean weekly work hours				
<30	5/356 (1.4)	4/299 (1.3)	9/655 (1.4)	.01
30-39	14/356 (3.9)	23/299 (7.7)	37/655 (5.6)	
40-49	67/356 (18.8)	79/299 (26.4)	146/655 (22.3)	
50-59	156/356 (43.8)	108/299 (36.1)	264/655 (40.3)	
≥60	114/356 (32.0)	85/299 (28.4)	199/655 (30.4)	
Current working status in medicine				
Full time	281/352 (79.8)	184/300 (61.3)	465/652 (71.3)	<.001
Part time	71/352 (20.2)	116/300 (38.7)	187/652 (28.7)	
Life experiences				
Spouse or partner’s employment				
Job type				
Physician	69/352 (19.6)	110/298 (36.9)	179/650 (27.5)	<.001
Not physician	131/352 (37.2)	134/298 (45.0)	265/650 (40.8)	
Does not currently have paid job	152/352 (43.2)	54/298 (18.1)	206/650 (31.7)	
If employed				
Full time	95/200 (47.5)	203/242 (83.9)	298/442 (67.4)	<.001
Part time	105/200 (52.5)	39/242 (16.1)	144/442 (32.6)	
Mean weekly work hours of spouse				
<20	31/200 (15.5)	11/244 (4.5)	42/444 (9.5)	<.001
20-29	28/200 (14.0)	10/244 (4.1)	38/444 (8.6)	
30-39	42/200 (21.0)	30/244 (12.3)	72/444 (16.2)	
40-49	57/200 (28.5)	103/244 (42.2)	160/444 (36.0)	
≥50	42/200 (21.0)	90/244 (36.9)	132/444 (29.7)	
Parental status				
Do you have children?				
Yes	330/359 (91.9)	256/302 (84.8)	586/661 (88.7)	.004
If so, how many children?				
Mean (SD)	2.6 (1.2)	2.2 (0.8)	2.4 (1.0)	<.001
Median (range)	2 (1-8)	2 (1-5)	2 (1-8)	
Age of youngest child, y				
<5	53/330 (16.1)	77/256 (30.1)	130/586 (22.2)	<.001
5-12	82/330 (24.8)	76/256 (29.7)	158/586 (27.0)	
13-18	48/330 (14.5)	50/256 (19.5)	98/586 (16.7)	
19-22	40/330 (12.1)	20/256 (7.8)	60/586 (10.2)	
≥23	107/330 (32.4)	33/256 (12.9)	140/586 (23.9)	
Responsibilities or duties in the home				
Hours/week spent on household or childcare duties or responsibilities, median (IQR)				
You: household	10 (7-19)	12 (9-20)	10 (8-20)	<.001
You: childcare	5 (0-14)	11 (4-33)	8 (1-20)	<.001
You: total (household + childcare)	16 (10-25)	25 (13-49)	20 (10-35)	<.001
Spouse or partner: household	20 (12-30)	10 (5-20)	15 (10-30)	<.001
Spouse or partner: childcare	10 (0-29)	10 (3-25)	10 (1-25)	.35
Spouse or partner: total (household + childcare)	33 (19-50)	20 (10-40)	30 (15-50)	<.001
% of Total time you spend (vs spouse of partner)	36 (25-47)	50 (42-67)	43 (32-52)	<.001
Caregiver for other(s): older adults, unwell individuals, and/or those with special needs	60/335 (17.9)	41/293 (14.0)	101/628 (16.1)	.18
If so, hours/week caregiving for others, median (IQR)	5 (2-8)	5 (2-8)	5 (2-8)	.67
Use of external professional help in the home for household or childcare or caregiving responsibilities	151/336 (44.9)	208/295 (70.5)	359/631 (56.9)	<.001
If so, hours/week professional help, median (IQR)	3 (2-8)	3 (2-17)	3 (2-10)	.28
Access to family and/or friends who can assist with household or caregiving duties				
Never	105/335 (31.3)	92/293 (31.4)	197/628 (31.4)	>.99
Occasionally or rarely	92/335 (27.5)	84/293 (28.7)	176/628 (28.0)	
Sometimes	57/335 (17.0)	47/293 (16.0)	104/628 (16.6)	
Often	29/335 (8.7)	22/293 (7.5)	51/628 (8.1)	
Most of the time	38/335 (11.3)	26/293 (8.9)	64/628 (10.2)	
Always	14/335 (4.2)	22/293 (7.5)	36/628 (5.7)	

Male respondents reported spending fewer hours per week addressing household or childcare duties than female respondents (household duties: median, 10 hours [IQR, 7-19 hours] vs 12 hours [IQR, 9-20 hours] and childcare duties: median, 5 hours [IQR, 0-14 hours] vs 11 hours [IQR, 4-33 hours]; *P* < .001) (Table 2). When asked how much time their spouse spends on these duties, male respondents reported a higher number of hours for household duties than female respondents (median, 20 hours [IQR, 12-30 hours] vs 10 hours [IQR, 5-20 hours]; *P* < .001); however, both male and female respondents reported that their spouse spent a median of 10 hours on childcare per week (IQR: spouses of male respondents, 0-29 hours; spouses of female respondents, 3-25 hours; *P* = .35). Considering the total hours of household or childcare duties (respondent and spouse combined), male respondents reported contributing to a median of 36% (IQR, 25%-47%) of the hours, whereas female respondents reported contributing to a median of 50% (IQR, 42%-67%) of the hours (*P* < .001). Caregiving in the home for older adults, unwell individuals, and/or those with special needs was reported by 101 of 628 respondents (16.1%) (median, 5 hours/week [IQR, 2-8 hours/week]), with no significant difference by gender (*P* = .67). Male respondents were less likely than female respondents to report using external professional help to assist with household or childcare or caregiving responsibilities (151 of 336 [44.9%] vs 208 of 295 [70.5%]; *P* < .001). Both male and female respondents reported using a median of 3 hours/week of such assistance (IQR: male respondents, 2-8 hours; female respondents, 2-17 hours; *P* = .28).

### Aim 1: Perceived Frequency of Spousal Support

Table 3 summarizes the frequency of spousal support reported by male and female physician respondents. Male and female respondents reported similar frequencies of career support from their spouse or partner, with 322 of 351 male physicians (91.7%) and 276 of 298 female physicians (92.6%) reporting frequent career support (often, most of time, always) over the last year from their spouse or partner. There was a lower frequency of support reported with respect to actually helping when busy: 75.9% of male physicians (264 of 348) reported that their spouse actually helped them often, most of the time, or always vs 68.7% of female physicians (204 of 297) (*P* = .03). Male and female respondents reported similar frequencies of their spouse expressing confidence in their ability to handle a situation (90.2% [581 of 644] at least often) and taking their side when they have a problem at work (88.5% [570 of 644] at least often). Overall mean (SD) composite spousal support scores were similar for male and female respondents (3.9 [0.9] vs 3.8 [0.9]; *P* = .35).

**Table 3.** Frequency of Spousal Support by Gender

Variable	No./total No. (%)			*P* value
	Male (n = 359)	Female (n = 302)	Total (N = 661)	
**Spousal support items (score)**				
Is supportive of my career				
Never (0)	1/351 (0.3)	0	1/649 (0.2)	.29
Occasionally or rarely (1)	5/351 (1.4)	4/298 (1.3)	9/649 (1.4)	
Sometimes (2)	23/351 (6.6)	18/298 (6.0)	41/649 (6.3)	
Often (3)	31/351 (8.8)	18/298 (6.0)	49/649 (7.6)	
Most of the time (4)	84/351 (23.9)	72/298 (24.2)	156/649 (24.0)	
Always (5)	207/351 (59.0)	186/298 (62.4)	393/649 (60.6)	
Mean (SD) score	4.3 (1.0)	4.4 (0.9)	4.4 (1.0)	
Offers to help me when I am busy				
Never (0)	3/348 (0.9)	6/296 (2.0)	9/644 (1.4)	.06
Occasionally or rarely (1)	25/348 (7.2)	31/296 (10.5)	56/644 (8.7)	
Sometimes (2)	54/348 (15.5)	59/296 (19.9)	113/644 (17.5)	
Often (3)	73/348 (21.0)	52/296 (17.6)	125/644 (19.4)	
Most of the time (4)	85/348 (24.4)	65/296 (22.0)	150/644 (23.3)	
Always (5)	108/348 (31.0)	83/296 (28.0)	191/644 (29.7)	
Mean (SD) score	3.5 (1.3)	3.3 (1.4)	3.4 (1.4)	
Helps me when I am busy				
Never (0)	4/348 (1.1)	5/297 (1.7)	9/645 (1.4)	.03
Occasionally or rarely (1)	29/348 (8.3)	33/297 (11.1)	62/645 (9.6)	
Sometimes (2)	51/348 (14.7)	55/297 (18.5)	106/645 (16.4)	
Often (3)	79/348 (22.7)	62/297 (20.9)	141/645 (21.9)	
Most of the time (4)	81/348 (23.3)	73/297 (24.6)	154/645 (23.9)	
Always (5)	104/348 (29.9)	69/297 (23.2)	173/645 (26.8)	
Mean (SD) score	3.5 (1.3)	3.3 (1.4)	3.4 (1.4)	
Expresses confidence in my ability to handle situation				
Never (0)	1/346 (0.3)	1/298 (0.3)	2/644 (0.3)	.73
Occasionally or rarely (1)	5/346 (1.4)	3/298 (1.0)	8/644 (1.2)	
Sometimes (2)	22/346 (6.4)	31/298 (10.4)	53/644 (8.2)	
Often (3)	40/346 (11.6)	27/298 (9.1)	67/644 (10.4)	
Most of the time (4)	96/346 (27.7)	70/298 (23.5)	166/644 (25.8)	
Always (5)	182/346 (52.6)	166/298 (55.7)	348/644 (54.0)	
Mean (SD) score	4.2 (1.0)	4.2 (1.1)	4.2 (1.0)	
Takes my side when I have a problem at work				
Never (0)	0	2/297 (0.7)	2/644 (0.3)	.77
Occasionally or rarely (1)	6/347 (1.7)	3/297 (1.0)	9/644 (1.4)	
Sometimes (2)	31/347 (8.9)	32/297 (10.8)	63/644 (9.8)	
Often (3)	72/347 (20.7)	59/297 (19.9)	131/644 (20.3)	
Most of the time (4)	146/347 (42.1)	112/297 (37.7)	258/644 (40.1)	
Always (5)	92/347 (26.5)	89/297 (30.0)	181/644 (28.1)	
Mean (SD) score	3.8 (1.0)	3.8 (1.0)	3.8 (1.0)	
Composite score, mean (SD)^a^	3.9 (0.9)	3.8 (0.9)	3.8 (0.9)	.35
**Key outcomes**				
Work-life integration (% satisfied)^b^	151/359 (42.1)	101/302 (33.4)	252/661 (38.1)	.02
Overall burnout (% at least weekly)^c^	130/354 (36.7)	112/301 (37.2)	242/655 (36.9)	.90

^a^ Composite score calculated as the mean of the 5 spousal support items.

^b^ Satisfaction with work-life integration defined as those responding with “strongly agree” or “agree” to “My work schedule leaves me enough time for my personal/family life.”

^c^ Overall burnout reflects those responding with “at least weekly” on either domain (emotional exhaustion or depersonalization). Denominator shown to reflect those with complete data for burnout items.

### Aim 2: Associations of Spousal Support and Gender With WLI Satisfaction and Burnout

The percentage of respondents satisfied with their WLI was higher for male vs female physicians (151 of 359 [42.1%] vs 101 of 302 [33.4%]; *P* = .02), but burnout rates were similar (130 of 354 [36.7%] vs 112 of 301 [37.2%]; *P* = .90) (Table 3). Table 4 displays results of multivariable logistic regression analyses examining associations of spousal support and gender with WLI satisfaction and burnout. Physicians who reported more frequent career support from their spouse or partner had higher odds of WLI satisfaction (OR, 1.50 [95% CI, 1.22-1.86]; *P* < .001) and lower odds of burnout (OR, 0.73 [95% CI, 0.61-0.87]; *P* < .001) independent of gender, age, race and ethnicity, weekly work hours, and weekly hours spent on household or childcare duties, as did physicians who reported more frequent expressions of confidence from a spouse or partner regarding their ability to handle situations (WLI: OR, 1.20 [95% CI, 1.01-1.44]; *P* = .04; burnout: OR, 0.83 [95% CI, 0.71-0.98]; *P* = .03). Overall frequency of spousal support, as measured by the composite spousal support score, was also associated with higher odds of WLI satisfaction (OR, 1.31 [95% CI, 1.06-1.63]; *P* = .01). None of the other forms of spousal support measured (offers to help when busy, actually helps when busy, takes my side) were associated with WLI or burnout in adjusted analyses. Among the spousal support items examined, career support showed the strongest association with WLI and burnout; the Figure displays the percentage of WLI and burnout by career support (combining the 3 lowest categories) and gender. Male physicians consistently had higher odds of WLI satisfaction than female physicians (OR, 1.95 [95% CI, 1.32-2.90]; *P* < .001), even after adjusting for spousal career support, age, race and ethnicity, weekly work hours, and weekly hours spent on household or childcare duties. The odds of burnout did not differ significantly based on gender (OR, 0.84 [95% CI, 0.57-1.23]; *P* = .37). There were no statistically significant interactions of spousal support with gender for either outcome (ie, associations of spousal support with WLI satisfaction or burnout did not differ significantly by gender).

**Table 4.** Logistic Regression Models Examining Associations of Spousal Support and Gender With WLI Satisfaction and Burnout^a^

Model and independent variable	WLI (satisfied)		Overall burnout	
	OR (95% CI)	*P* value	OR (95% CI)	*P* value
Model 1a (unadjusted)				
Is supportive of my career	1.48 (1.24-1.80)	<.001	0.71 (0.60-0.83)	<.001
Model 1b (adjusted)				
Is supportive of my career	1.50 (1.22-1.86)	<.001	0.73 (0.61-0.87)	<.001
Gender (male vs female)	1.95 (1.32-2.90)	<.001	0.84 (0.57-1.23)	.37
Age (1-category increase)	0.83 (0.69-1.01)	.06	1.00 (0.83-1.21)	.99
Underrepresented race and ethnicity (yes vs no)	1.21 (0.62-2.32)	.57	0.75 (0.37-1.44)	.40
Weekly work hours (1-category increase)	0.47 (0.38-0.57)	<.001	1.63 (1.33-2.02)	<.001
Weekly hours spent on household or childcare duties (1-h increase)	0.99 (0.98-1.00)	.02	1.01 (1.00-1.01)	.21
Model 2a (unadjusted)				
Offers to help me when I am busy	1.12 (1.00-1.26)	.06	0.98 (0.87-1.11)	.78
Model 2b (adjusted)				
Offers to help me when I am busy	1.13 (0.99-1.30)	.07	0.98 (0.86-1.11)	.75
Gender (male vs female)	1.77 (1.21-2.63)	.004	0.90 (0.62-1.31)	.58
Age (1-category increase)	0.84 (0.70-1.02)	.07	1.01 (0.84-1.22)	.89
Underrepresented race and ethnicity (yes vs no)	1.20 (0.61-2.31)	.59	0.78 (0.39-1.51)	.48
Weekly work hours (1-category increase)	0.46 (0.37-0.57)	<.001	1.63 (1.33-2.01)	<.001
Weekly hours spent on household or childcare duties (1-h increase)	0.99 (0.98-1.00)	.01	1.01 (1.00-1.02)	.10
Model 3a (unadjusted)				
Helps me when I am busy	1.11 (0.99-1.25)	.09	0.95 (0.84-1.07)	.39
Model 3b (adjusted)				
Helps me when I am busy	1.11 (0.97-1.28)	.13	0.96 (0.84-1.09)	.49
Gender (male vs female)	1.78 (1.21-2.63)	.004	0.91 (0.62-1.33)	.62
Age (1-category increase)	0.84 (0.69-1.01)	.07	1.01 (0.83-1.21)	.95
Underrepresented race and ethnicity (yes vs no)	1.15 (0.59-2.20)	.67	0.77 (0.38-1.48)	.44
Weekly work hours (1-category increase)	0.47 (0.38-0.57)	<.001	1.64 (1.34-2.02)	<.001
Weekly hours spent on household or childcare duties (1-h increase)	0.99 (0.98-1.00)	.007	1.01 (1.00-1.02)	.11
Model 4a (unadjusted)				
Expresses confidence in my ability to handle situation	1.19 (1.02-1.40)	.03	0.83 (0.71-0.97)	.02
Model 4b (adjusted)				
Expresses confidence in my ability to handle situation	1.20 (1.01-1.44)	.04	0.83 (0.71-0.98)	.03
Gender (male vs female)	1.83 (1.25-2.72)	.002	0.89 (0.61-1.30)	.54
Age (1-category increase)	0.83 (0.69-1.00)	.05	1.01 (0.84-1.22)	.94
Underrepresented race and ethnicity (yes vs no)	1.15 (0.59-2.20)	.67	0.79 (0.39-1.51)	.49
Weekly work hours (1-category increase)	0.47 (0.38-0.57)	<.001	1.64 (1.33-2.02)	<.001
Weekly hours spent on household or childcare duties (1-h increase)	0.99 (0.98-1.00)	.01	1.01 (1.00-1.02)	.16
Model 5a (unadjusted)				
Takes my side when I have a problem at work	1.10 (0.94-1.29)	.24	0.90 (0.77-1.06)	.21
Model 5b (adjusted)				
Takes my side when I have a problem at work	1.07 (0.89-1.28)	.47	0.94 (0.79-1.11)	.46
Gender (male vs female)	1.82 (1.24-2.69)	.003	0.88 (0.61-1.29)	.52
Age (1-category increase)	0.83 (0.69-1.00)	.06	1.01 (0.84-1.22)	.93
Underrepresented race and ethnicity (yes vs no)	1.22 (0.63-2.34)	.55	0.78 (0.39-1.50)	.47
Weekly work hours (1-category increase)	0.47 (0.38-0.58)	<.001	1.62 (1.32-2.00)	<.001
Weekly hours spent on household or childcare duties (1-h increase)	0.99 (0.98-1.00)	.006	1.01 (1.00-1.02)	.11
Model 6a (unadjusted)				
Composite spousal support score	1.31 (1.09-1.58)	.005	0.83 (0.69-0.99)	.04
Model 6b (adjusted)				
Composite spousal support score	1.31 (1.06-1.63)	.01	0.83 (0.69-1.02)	.07
Gender (male vs female)	1.81 (1.23-2.68)	.003	0.90 (0.61-1.31)	.57
Age (1-category increase)	0.84 (0.70-1.02)	.08	1.00 (0.83-1.20)	.96
Underrepresented race and ethnicity (yes vs no)	1.14 (0.59-2.18)	.69	0.78 (0.39-1.49)	.46
Weekly work hours (1-category increase)	0.47 (0.38-0.57)	<.001	1.63 (1.33-2.02)	<.001
Weekly hours spent on household or childcare duties (1-h increase)	0.99 (0.98-1.00)	.01	1.01 (1.00-1.02)	.16

Abbreviations: OR, odds ratio; WLI, work-life integration.

^a^ Adjusted for age, underrepresented race and ethnicity, weekly work hours, and weekly hours spent on household or childcare duties. No statistically significant interactions between spousal support and gender were found within any model (all *P* > .05). ORs for support items are shown for a 1-level increase on a 6-point scale from never (0) to always (5).

**Figure.**
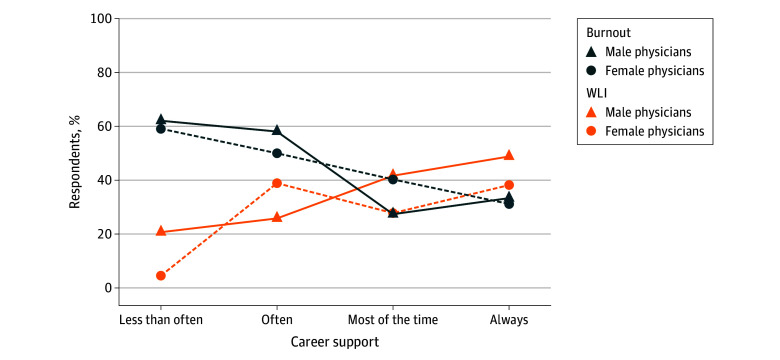
Work-Life Integration (WLI) and Burnout by Career Support and Gender

## Discussion

In this cross-sectional, multispecialty survey of 661 married or partnered academic faculty physicians, more frequent career support from a spouse or partner was associated with higher odds of WLI satisfaction and lower odds of burnout, independent of gender and other covariates. Male physicians had higher odds of WLI satisfaction than female physicians, even after adjusting for spousal career support, age, race and ethnicity, weekly work hours, and weekly hours spent on childcare and household duties. Male physicians reported more weekly work hours and were more likely to work full time than female physicians. They also spent fewer hours per week addressing household or childcare duties and reported more frequent actual help from their spouse or partner than female physicians. The frequency of other forms of spousal support was similar between male and female physicians. Taken together, these findings build on prior research in at least 3 substantive ways.

First, we found that both male and female respondents reported frequent spousal support overall. Prior research postulates that gender-role socialization can lead to a “support gap hypothesis”^13^ whereby women may receive less spousal support compared with their male counterparts. Our findings partially support this hypothesis in that male physicians reported more frequent offers to help and actual help when they were busy than female physicians—possibly because male physicians were less likely to have a physician spouse and more likely to have a spouse working part time or at home. No statistically significant differences were reported between male and female physicians regarding career support from their spouse or partner, however, which may reflect evolving societal norms around women working professionally.

Second, we studied associations of spousal support with WLI and burnout, whereas most literature regarding physician WLI and burnout focuses on individual and institutional contributors to these variables. The associations we observed of spousal support with these outcomes highlight that other factors outside work may also be associated with physician burnout and WLI satisfaction. Although factors extrinsic to the workplace cannot be directly addressed by health systems, this finding is important, as addressing WLI and burnout at the personal and organizational levels alone may not be adequate for physicians with suboptimal supports outside work. The job demands–resources model offers a potential conceptual lens to better understand these findings.^25^ This model assumes that every occupation has unique demands and requires tailored resources and supports. Prior research has also demonstrated that excessive personal and professional demands can have an adverse association with employee well-being, burnout, and work performance, whereas personal and professional resources can improve employee motivation and health.^26,27^ However, the role of potential interventions, such as marriage counseling, is unclear and merits additional study. Therefore, a multifaceted approach including other interventions to enhance well-being may improve WLI and reduce burnout. This may be especially relevant for spouses who have different occupations, as spouses in a dual-physician marriage may have better spousal support thanks to empathy born from shared professional experiences.^16,28^

Third, we found that male physicians had higher odds of WLI satisfaction than female physicians, even after adjusting for various forms of spousal support (along with age, race and ethnicity, weekly work hours, and weekly hours spent on household or childcare duties). This finding suggests that frequency of spousal support does not fully account for the gender-based differences in WLI satisfaction that we observed and that have been reported in prior survey research.^23^ Other factors that could be associated with gender disparities in WLI satisfaction include the fact that female physicians may face prejudice and microaggressions at work (eg, medical cultures and structures against women, undervalued contributions, longer hours spent on patient care [including electronic health record time]) and increased work-life expectations during COVID-19 (homeschooling and/or caregiving).^15,26,29,30,31,32,33,34,35,36^

### Limitations

This study has some limitations. First, cross-sectional surveys cannot establish causal relationships. Second, study participants were from a single US academic health center located in the Midwest and identified as male or female, and most did not belong to URM racial or ethnic groups, which could affect the generalizability of our results. It is possible that respondents at high risk of burnout were not well represented in this group, which may represent better supported individuals who have survived to this point. Third, this study does not account well for the unique challenges of physicians who have separated households (ie, those living separately from a partner and/or children some or all of the time). Fourth, we measured weekly work hours and weekly hours spent on household or childcare duties via self-report, which may not accurately reflect actual work and caregiving hours. Fifth, we prioritized measures of spousal support and work-life experiences of physicians to illuminate the potential relevance of these factors to WLI and burnout and did not measure other well-established factors associated with burnout^37^ (eg, work control, meaning in work) to keep the survey length manageable, which risks the possibility of omitted variable bias. Sixth, there could be variability regarding how “frequent spousal support” was defined by respondents, as well as favorability bias or reluctance to characterize one’s spouse as unsupportive. Future research should include more ethnically, racially, and geographically diverse faculty physicians with varied gender identities and family structures that more fully reflect modern society.

## Conclusions

The results of this cross-sectional survey study highlight the potential importance of career support from a spouse or partner for WLI and physician well-being and suggest that spousal support, weekly work hours, and weekly hours spent on household and childcare duties do not fully account for lower WLI satisfaction among female physicians. Additional longitudinal studies are needed to better understand gendered physician WLI experiences and determine if increasing career support for physicians outside work leads to greater satisfaction with WLI and reduced burnout.

## Notes

Supplement 1. **eAppendix. **Institutional Survey

Supplement 2.
Data Sharing Statement
