# Supplementary material for: Spousal Support and Physician Work-Life Integration and Burnout
Source: JAMA Netw Open. 2025 May 9;8(5):e259507. doi: 10.1001/jamanetworkopen.2025.9507 (PMC12065037; doi:10.1001/jamanetworkopen.2025.9507)
Supplement: Supplement 1. — eAppendix. Institutional Survey [file jamanetwopen-e259507-s001.pdf]

Supplementary Online Content

O'Sullivan CC, Jenkins S, Leep Hunderfund AN, Ruddy KJ, West CP, Marshall AL. Spousal support and physician work-life integration and burnout. *JAMA Netw Open.* 2025;8(5):e259507. doi:10.1001/jamanetworkopen.2025.9507

eAppendix. Institutional Survey

This supplementary material has been provided by the authors to give readers additional information about their work.

© 2025 O'Sullivan CC et al. *JAMA Network Open.*

eAppendix. Institutional Survey

Survey Research Center

Default Question Block

Please provide your name and email address.

This information will NOT be connected to your survey responses and will ONLY be used for the aforementioned purpose.

First Name:	
Last Name:	
Email:	

Submit

References

[1]Shanafelt TD, Boone S, Tan L, Dyrbye LN, Sotile W, Satele D, West CP, Sloan J, Oreskovich MR. **Burnout and satisfaction with work-life balance among US physicians relative to the general US population**. Arch Intern Med. 2012 Oct 8;172(18):1377-85. doi: 10.1001/archinternmed.2012.3199. PMID: 22911330.

[2]Li-Sauerwine S, Rebillot K, Melamed M, Addo N, Lin M. A **2-Question Summative Score Correlates with the Maslach Burnout Inventory**. West J Emerg Med. 2020 Apr 21;21(3):610-617. doi: 10.5811/westjem.2020.2.45139. PMID: 32421508; PMCID: PMC7234685

[3]Dehle C, Larsen D, Landers JE. **Support in Intimate Relationships Rating Scale (SIRRS). Social Support in Marriage**, The American Journal of Family Therapy 2001, 29:4, 307-324, DOI: 10.1080/01926180126500.

THANK YOU FOR COMPLETING THE SURVEY!

Please click SUBMIT to record your answers.

© 2025 O’Sullivan CC et al. *JAMA Network Open.*

Section 1: Demographics

The Association of Spousal Status, Support, and Non-Clinical Responsibilities with Physician Work-Life Integration, Burnout, and Workforce Attrition: A Survey Study

Section 1: Demographics:

Age:

- ◯ Less than 30 years old
- ◯ 30 to 39 years old
- ◯ 40 to 49 years old
- ◯ 50 to 59 years old
- ◯ 60 years old or older

Race: (Select all that apply.)

- ☐ American Indian or Alaska Native
- ☐ Asian
- ☐ Black or African American
- ☐ Native Hawaiian or Other Pacific Islander
- ☐ White
- ☐ Other, please specify: ________
- ☐ Prefer not to answer

Ethnicity:

© 2025 O'Sullivan CC et al. *JAMA Network Open.*

- Hispanic or Latino or Spanish Origin
- Not Hispanic or Latino or Spanish Origin
- Prefer not to answer

Gender Identity:

- Male
- Female
- Other, please specify:
- Prefer not to answer

Years in practice:

- Less than 10 years
- 10 to 19 years
- 20 years or more

Please select your specialty:

- Allergy and Immunology
- Anesthesiology
- Colon and Rectal Surgery
- Dermatology
- Emergency Medicine
- Family Medicine
- Internal Medicine and other medicine subspecialties, please specify:
- Medical Genetics and Genomics
- Neurological Surgery
- Neurology
- Nuclear Medicine
- Obstetrics and Gynecology
- Ophthalmology

© 2025 O'Sullivan CC et al. *JAMA Network Open*.

- ○ Orthopedic Surgery
- ○ Pathology
- ○ Pediatrics and other pediatric subspecialties, please specify: ______
- ○ Physical Medicine and Rehabilitation
- ○ Plastic Surgery
- ○ Preventative Medicine
- ○ Psychiatry
- ○ Radiation Oncology
- ○ Radiology
- ○ Surgery and other surgical subspecialties, please specify: ______
- ○ Thoracic Surgery
- ○ Urology

Mean total hours/week spent on work-related responsibilities:

- ○ Less than 30 hours
- ○ 30 to 39 hours
- ○ 40 to 49 hours
- ○ 50 to 59 hours
- ○ 60 hours or more

Which of the following best describes your current relationship status?

- ○ Single
- ○ Married
- ○ Partnered
- ○ Separated/divorced
- ○ Widowed/widower

Do you have a spouse/partner currently?

- ○ Yes

© 2025 O'Sullivan CC et al. *JAMA Network Open*.

○ No

Do you have children?

○ Yes
○ No

How many children do you have?

[ ]

What is your youngest child's age?

○ Less than 5 years old
○ 5 to 12 years old
○ 13 to 18 years old
○ 19 to 22 years old
○ 23 years old or older

Section 2: Work-Life Integration

Section 2: Work-Life Integration

Please select **one response** from the options listed below:

My work schedule leaves me enough time for my personal/family life.[1]

○ Strongly agree
○ Agree
○ Neutral
○ Disagree
○ Strongly disagree

© 2025 O'Sullivan CC et al. *JAMA Network Open.*

Section 3. Burnout

Section 3. Burnout

Please indicate how often you have had the following feelings about your job.

I feel burned out from my work.[2]

- Never
- A few times a year or less
- Once a month or less
- A few times a month
- Once a week
- A few times a week
- Every day

I have become more callous toward people since I took this job.[2]

- Never
- A few times a year or less
- Once a month or less
- A few times a month
- Once a week
- A few times a week
- Every day

Section 4. Workforce Attrition

Section 4. Workforce Attrition

What is your current working status in medicine?

- Full-time (1.0 FTE)

© 2025 O'Sullivan CC et al. *JAMA Network Open.*

◯ Part-time (<1.0 FTE)

Have you ever considered working part-time?

◯ Yes

◯ No

Regarding reasons for your interest in part-time work: (Select all that apply.)

☐ Increased flexibility

☐ Increased autonomy (more freedom to make work/career-related decisions and fulfill professional goals)

☐ Increased control over the workplace (schedule, pace, interruptions)

☐ Pursuit of other professional interests (teaching, research)

☐ Pursuit of separate employment opportunities outside of Mayo Clinic

☐ Improved work-life integration

☐ Family commitments (spouse, children, elder care, etc.)

☐ Other, please specify: ________

Have you considered leaving academic medicine over the next 2 years?

◯ Yes

◯ No

Regarding reasons why you have considered leaving academic medicine: (Select all that apply.)

☐ Insufficient mentorship

☐ Challenges in career advancement (leadership opportunities)

☐ Challenges in academic promotion

☐ Lack of protected time for research

☐ Lack of protected time for education/teaching

☐ Lack of protected time for clinical administrative tasks (portal messages, pre-authorizations, etc.)

☐ Salary concerns

© 2025 O'Sullivan CC et al. *JAMA Network Open.*

- ☐ Difficulty balancing clinical, research, and/or educational responsibilities
- ☐ Personal/family reasons
- ☐ Gender inequities and/or discrimination
- ☐ Racial/ethnic inequities and/or discrimination
- ☐ Other, please specify: ____________

Section 5: Spousal/Partner Support

<u>Section 5: Spousal/Partner Support</u>

Over the past one year, my spouse/partner has been supportive of my career.[3]

- ○ Never
- ○ Occasionally/rarely
- ○ Sometimes
- ○ Often
- ○ Most of the time
- ○ Always

Regarding my spouse/partner's employment:[3]

- ○ My spouse/partner has a paid job as a physician
- ○ My spouse/partner has a paid job but is not a physician
- ○ My spouse/partner does not currently have a paid job

Do they:[3]

- ○ Work full-time
- ○ Work part-time

Over the past one year, my spouse/partner's career has taken priority over mine.[3]

- ○ Never

© 2025 O'Sullivan CC et al. *JAMA Network Open.*

- Occasionally/rarely
- Sometimes
- Often
- Most of the time
- Always

Each week, my spouse/partner works in a professional capacity (paid job) for an average of:[3]

- Less than 20 hours
- 20 to 29 hours
- 30 to 39 hours
- 40 to 49 hours
- 50 hours or more

Whenever I am busy with professional and household responsibilities, my spouse/partner offers to help me (e.g., offers to do my chores).[3]

- Never
- Occasionally/rarely
- Sometimes
- Often
- Most of the time
- Always

Whenever I am busy with professional and household responsibilities, my spouse/partner helps me (e.g., does my chores).[3]

- Never
- Occasionally/rarely
- Sometimes
- Often
- Most of the time
- Always

© 2025 O'Sullivan CC et al. *JAMA Network Open.*

When I have a problem at work, my spouse/partner expresses confidence in my ability to handle a situation.[3]

- ◯ Never
- ◯ Occasionally/rarely
- ◯ Sometimes
- ◯ Often
- ◯ Most of the time
- ◯ Always

When I have a problem at work, my spouse/partner takes my side when discussing my situation.[3]

- ◯ Never
- ◯ Occasionally/rarely
- ◯ Sometimes
- ◯ Often
- ◯ Most of the time
- ◯ Always

Section 6. Household and Caregiving Duties

Section 6. Household and Caregiving Duties

How many hours each week, on average, do you spend addressing household duties/responsibilities?

[ ] hours

How many hours each week, on average, do you spend addressing childcare responsibilities?

[ ] hours

© 2025 O’Sullivan CC et al. *JAMA Network Open*.

If you have a partner/spouse, how many hours each week, on average, do they spend addressing household duties/responsibilities?

[ ] hours

If you have a partner/spouse, how many hours each week, on average, do they spend addressing childcare responsibilities?

[ ] hours

Over the past one year, have you used external professional help in the home to assist with household and/or childcare/caregiving responsibilities?

- Yes
- No

On average, how many hours a week do you use external professional help in the home to assist with household and/or childcare/caregiving responsibilities?

[ ] hours

Outside of caring for your children (if applicable), are you a caregiver for other children (e.g., grandchildren), or an individual who is elderly, unwell, and/or has special needs?

- Yes
- No

How many hours each week, on average, do you spend providing care for the individual(s) in question?

[ ] hours

Over the past one year, I have had access to family and/or friends nearby who can assist me with household and/or caregiving duties when needed.

- Never
- Occasionally/rarely
- Sometimes

© 2025 O'Sullivan CC et al. *JAMA Network Open.*

- Often
- Most of the time
- Always

Section 7: Further Research Opportunities

Section 7: Further Research Opportunities

If a follow-up interview or focus group study is conducted to better understand work-life integration experiences, would you like to receive an email invitation to participate?

- Yes
- No

Submit

References

[1]Shanafelt TD, Boone S, Tan L, Dyrbye LN, Sotile W, Satele D, West CP, Sloan J, Oreskovich MR. **Burnout and satisfaction with work-life balance among US physicians relative to the general US population**. Arch Intern Med. 2012 Oct 8;172(18):1377-85. doi: 10.1001/archinternmed.2012.3199. PMID: 22911330.

[2]Li-Sauerwine S, Rebillot K, Melamed M, Addo N, Lin M. A **2-Question Summative Score Correlates with the Maslach Burnout Inventory**. West J Emerg Med. 2020 Apr 21;21(3):610-617. doi: 10.5811/westjem.2020.2.45139. PMID: 32421508; PMCID: PMC7234685

[3]Dehle C, Larsen D, Landers JE. **Support in Intimate Relationships Rating Scale (SIRRS). Social Support in Marriage**, The American Journal of Family Therapy 2001, 29:4, 307-324, DOI: 10.1080/01926180126500.

THANK YOU FOR COMPLETING THE SURVEY!

Please click SUBMIT to record your answers.

© 2025 O'Sullivan CC et al. *JAMA Network Open.*
