# Supplementary material for: Spousal Support and Physician Work-Life Integration and Burnout
Source: JAMA Netw Open. 2025 May 9;8(5):e259507. doi: 10.1001/jamanetworkopen.2025.9507 (PMC12065037; doi:10.1001/jamanetworkopen.2025.9507)
Supplement: Supplement 2. — Data Sharing Statement [file jamanetwopen-e259507-s002.pdf]

Data Sharing Statement

O'Sullivan. Spousal Support and Physician Work-Life Integration and Burnout. *JAMA Netw Open*. Published May 09, 2025. doi:10.1001/jamanetworkopen.2025.9507

Data
Data available: No
